# Colonic Adenosquamous Carcinoma: A Single-Center Review of Patient Clinicopathologic Characteristics, Genetics, and Clinical Outcomes

**DOI:** 10.3390/cancers16152641

**Published:** 2024-07-25

**Authors:** David A. Lieb, Hannah M. Thompson, Floris S. Verheij, Jinru Shia, Francisco Sanchez-Vega, Georgios Karagkounis, Maria Widmar, Iris H. Wei, J. Joshua Smith, Garrett M. Nash, Martin R. Weiser, Philip B. Paty, Andrea Cercek, Leonard B. Saltz, Julio Garcia-Aguilar, Emmanouil Pappou

**Affiliations:** 1 Department of Colorectal Surgery, Memorial Sloan-Kettering Cancer Center, New York, NY 10065, USAweii@mskcc.org (I.H.W.); smithj5@mskcc.org (J.J.S.); weiser1@mskcc.org (M.R.W.);; 2 Army Medical Department (AMEDD) Student Detachment, US Army Medical Center of Excellence, JBSA Fort Sam Houston, Fort Sam Houston, TX 78234, USA; 3 Department of Pathology, Memorial Sloan-Kettering Cancer Center, New York, NY 10065, USA; 4 Department of Computational Biology, Memorial Sloan-Kettering Cancer Center, New York, NY 10065, USA; 5 Department of Medicine, Memorial Sloan-Kettering Cancer Center, New York, NY 10065, USA

**Keywords:** adenosquamous colon cancer, colon cancer, adenosquamous

## Abstract

**Simple Summary:**

Colonic adenosquamous carcinoma is an extremely rare subtype of colon cancer, making its study and characterization challenging. Here, we demonstrate the clinical properties of a cohort of patients with this rare subtype, characterized by a relatively young age at the time of diagnosis, right-sided predominance, an advanced stage at the time of diagnosis, frequent recurrence, and poor overall survival. All of these add further to the limited body of literature highlighting the more aggressive nature of colonic adenosquamous carcinoma, particularly when compared to adenocarcinoma.

**Abstract:**

(1) Background: Adenosquamous carcinoma (ASC) is a rare subtype of colon cancer. Its rarity makes characterization challenging, although colonic ASC is believed to present at more advanced stages and have worse outcomes versus adenocarcinoma. This study aims to characterize the clinicopathological characteristics and clinical outcomes of colonic ASC. (2) Methods: This is a single-center, retrospective review of patients diagnosed with colonic ASC from 2000 to 2020. Data extracted included patient demographics, staging at diagnosis, tumor clinicopathologic and genetic characteristics, and clinical outcomes. (3) Results: Among 61,126 patients with colorectal cancer, 13 (0.02%) had colonic ASC, with a mean age at diagnosis of 48.7 years. The cecum/ascending colon was the most common primary site (6/13, 46.2%), and all except one patient was diagnosed with Stage III or IV disease. Among the eight patients with mismatch repair genetics available, only one was mismatch repair deficient. Eleven patients (84.6%) underwent surgery, and 11 likewise received some form of chemotherapy. Recurrence occurred in 7 of 13 patients (53.8%), and the overall five-year survival rate was 38.5%. The median survival rate was 39.4 months overall (30.5 months for Stage III, 23.7 months for Stage IV). (4) Conclusions: Overall, colonic ASC is rare, and this cohort of colonic ASC patients demonstrated advanced stage at diagnosis, frequent recurrence, and poor overall survival. Additional research remains to compare these characteristics with those of comparably staged adenocarcinoma and to develop specific management recommendations.

## 1. Introduction

Colorectal cancer (CRC) is one of the most prominent malignancies in the world, with an estimated 147,950 newly diagnosed cases and 53,200 associated deaths for the year 2020 in the United States [1]. Although most CRC is adenocarcinoma, other subtypes exist, including adenosquamous carcinoma (ASC), a rare subtype that comprises less than 1% of all gastrointestinal cancers and contains histological features of both adenocarcinoma and squamous cell carcinoma. The existing literature on gastric and pancreatic cancers suggests that, compared to adenocarcinoma, ASC presents at a more advanced stage at the time of diagnosis and is associated with worse overall survival, even among comparably staged patients [2,3]. Colonic ASC is especially rare and comprises less than 0.1% of all diagnosed CRCs. Due to its rarity, most of the literature on this entity consists of case reports and small case series.

That said, some patterns have emerged in the few large-scale studies that have been published, many of which are consistent with findings regarding ASC in other organs. With regards to demographics, ASC tends to present predominantly in Caucasians, with many studies showing a slight female predominance. ASC of the colon most typically arises in the ascending colon, although Cagir et al. found that among a patient cohort with colonic ASC, the descending colon was more common as a primary site [4]. Consistent with other GI ASC malignancies, colonic ASC is typically diagnosed at a more advanced stage and at a younger mean age of diagnosis compared to adenocarcinoma [5]. Additionally, while the more advanced stage at the time of diagnosis may be responsible for worse outcomes, Khan et al. found that even among comparably staged colon cancers, the overall 5-year survival rate was worse for ASC vs. adenocarcinoma [6].

While the literature has provided insight regarding the clinical course of colonic ASC, in-depth studies remain challenging to complete, including specific treatment recommendations compared to colonic adenocarcinoma. With these gaps of knowledge in mind, this study sought to examine the demographics and clinicopathologic characteristics of patients with colonic ASC, to include treatment courses and outcomes. We predicted that colonic ASC would be associated with a greater rate of recurrence and a lower 5-year survival rate compared to colonic adenocarcinoma with similar patient demographics, stage at diagnosis, and treatment.

## 2. Materials and Methods

This study was a retrospective, single-center review (Memorial Sloan-Kettering Cancer Center, New York, NY, USA) of patients with colonic adenosquamous carcinoma (ASC). The study protocol was reviewed and approved by our institution’s Institutional Review Board (IRB Approval number 16-1265). Within the institutional databank of patients with colon cancer, we screened patients with colonic ASC who were identified using ICD-O Histology codes M8560/3 (Adenosquamous carcinoma) and M8075/3 (Adenoid Squamous Cell Carcinoma), from 1 January 2000 through to 1 December 2021. Patients were eligible for inclusion if ASC was identified either on the initial biopsy of the primary tumor or upon final surgical pathology. Exclusion criteria included incomplete clinical information, anorectal ASC (which represents a separate clinical entity), or the final pathology was determined to not be ASC (e.g., pure squamous cell carcinoma).

After identifying eligible patients, a chart review was performed to extract pertinent clinical and demographic information, including age, gender, race/ethnicity, smoking history, prior medical history, and American Joint Committee on Cancer (AJCC) stage at the time of diagnosis. Any available pathology and genetic tumor information was additionally reviewed. We focused primarily on mismatch repair (MMR) proficiency data and Memorial Sloan Kettering Integrated Mutation Profiling of Actionable Cancer Targets (MSK-IMPACT), a whole exome sequencing tool at our institution that captures the copy number and mutational burden of several key oncogenes and tumor suppressor genes (e.g., TP53, PTEN, and KRAS). Finally, patients’ records were reviewed to identify any chemotherapy or surgery performed, recurrence, disease-free survival, and overall survival rate.

Patients were followed through on 31 March 2022. The original intent of this study was to match patients with colonic ASC with patients with colonic adenocarcinoma with comparable demographics and stages at the time of diagnosis, with a subsequent comparison of clinical outcomes. However, after the final number of patients with ASC was determined following initial screening, the statistical power was found to be too low to draw any meaningful inferences. As such, this matching and subsequent analysis was not performed.

## 3. Results

**Figure 1:**
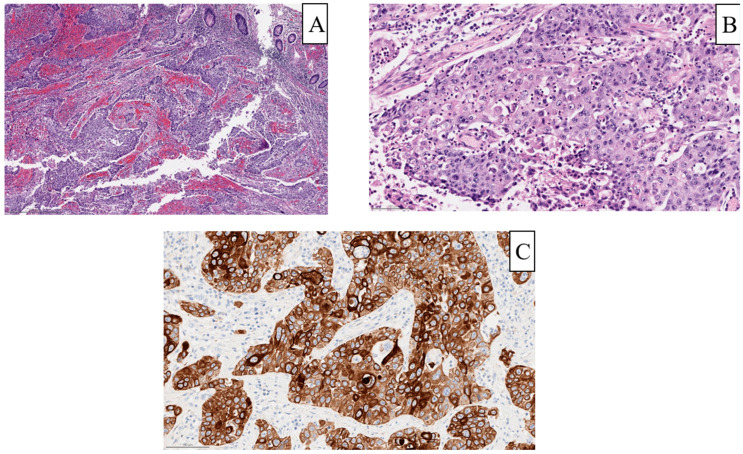
Sample pathology from a patient within the cohort with final pathology of poorly differentiated carcinoma with squamous differentiation. (**A**) Low power view (scale bar, 500 µm) showing irregular nests of tumor and little evidence of gland formation or mucin production. (**B**) High power view (scale bar, 100 µm) showing more prominent features of squamous differentiation, such as intra-cellular bridges. (**C**) Immunohistochemical staining for high molecular weight keratin showing diffuse positive staining indicative of squamous differentiation.

Among 61,126 total patients diagnosed with colorectal cancer in the study period, screening initially identified 28 patients with ASC, of whom 20 had sufficient clinical data for analysis. After excluding anorectal cases of ASC, the final cohort consisted of 13 cases of colonic ASC (incidence 0.02%). Figure 1 illustrates the histological features of ASC from one such patient in this cohort, with both squamous and glandular elements on their pathology. The average age of the cohort at the time of diagnosis was 48.7 years, with 7 of 13 patients (58.3%) being younger than 50, and the majority were male (8/13, 61.5%) and white (Table 1).

**Figure 2:**
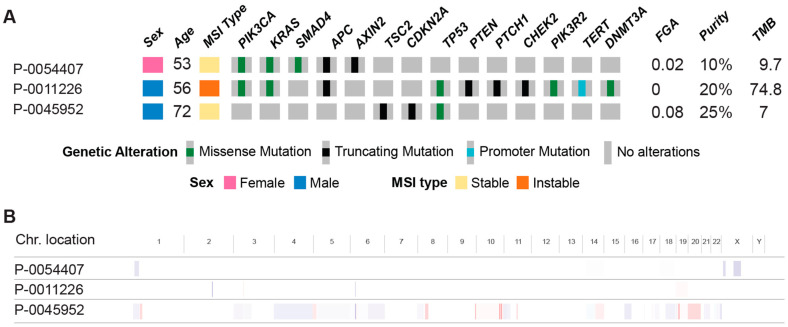
Genetic profile of 3 patients with MSK-IMPACT, highlighting specific mutations affecting selected oncogenes and tumor-suppressor genes (**A**) and chromosome location (**B**). (MSI = microsatellite instability, FGA = fraction of genome altered by copy number changes, and TMB = tumor mutational burden).

The ascending colon and cecum were the most common primary tumor locations, collectively comprising 46.2% of cases, followed by the transverse colon (including the splenic flexure). All but one patient presented with Stage III or IV disease, with six presenting at Stage III and six presenting at Stage IV at the time of diagnosis; the remaining patient presented at Stage II (Table 2). The mismatch repair (MMR) status was only available for eight patients, with one mismatch repair deficient patient and the remaining seven being mismatch repair proficient. MSK-IMPACT data were available for three patients within our cohort (Figure 2). One patient was mismatch repair deficient and had a high mutation burden, with mutations identified in multiple oncogenes. Conversely, the other two patients who were mismatch repair proficient had a comparatively lower tumor burden and copy number changes. Four specific genes were found to have mutations in two of these three patients: PIK3CA, KRAS, APC, and TP53.

Table 3 lists the clinical management and outcomes of the cohort. Eleven patients (84.6%) underwent surgical management, and 11 patients received some form of chemotherapy. Among the 11 patients undergoing chemotherapy, eight received adjuvant chemotherapy alone, two received neoadjuvant and adjuvant chemotherapy, and one received neoadjuvant chemotherapy alone. All patients undergoing chemotherapy, adjuvant and neoadjuvant, received 5-fluorouracil, folinic acid, and oxaliplatin (FOLFOX). Three patients additionally received bevacizumab and one patient additionally received floxuridine and irinotecan.

**Figure 3:**
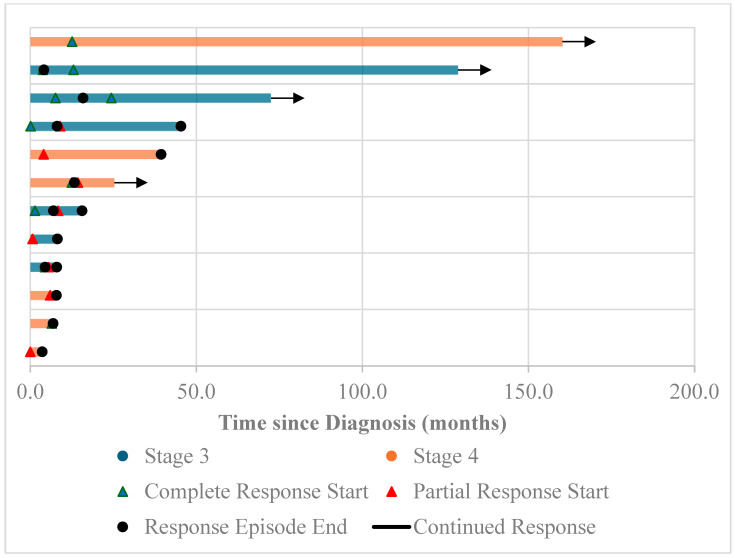
Swimmer’s plot of Stage III and IV adenosquamous carcinoma (ASC) patients in the cohort, highlighting the frequency of recurrences within the cohort.

Following diagnosis, the median follow-up period was 15.6 months. Recurrence was common, with seven patients (53.8%) having some type of recurrence (Figure 3). Among these, four patients had locoregional recurrence, two patients had distal recurrence, and one patient had both locoregional and distal recurrence. The overall five-year survival rate was 38.5%, with mortality in 8/13 patients; this was lower for Stage III and IV patients, each of which had a 5-year survival rate of 33.3%. The overall median survival rate was 39.4 months; this again was lower for patients initially diagnosed with Stage III (30.5 months) and Stage IV (23.7 months) disease.

## 4. Discussion

Adenosquamous carcinoma (ASC) is defined by the presence of glandular and squamous elements upon pathology. It is an extremely rare subtype of colon cancer, with this study identifying colonic ASC in only 0.02% of all colon cancers from our institution. This cohort was likewise relatively young overall, with an average age of 48.7 years at the time of diagnosis. The cecum/ascending colon represented the most common primary site (46%), followed by the transverse (31%) and the descending/sigmoid colon (23%). All but one patient presented with either Stage III or IV disease, with all patients being treated appropriately based on AJCC guidelines. Genetic profiling revealed only one patient with confirmed MMR deficiency (7% overall); aside from this patient, the mutational burden was overall low in patients with complete genetic sequencing available. Despite optimal management, recurrence occurred in 53.8% of patients, with a five-year survival of 38.5%, and a median survival of 39.4 months. While these findings are consistent with similar findings in the literature, they nonetheless prove an invaluable clinical characterization given the limited number of published studies available. Certain findings in this cohort are worthy of further discussion.

Consistent with previous studies, most patients in this cohort were Caucasian and male, which contrasts with studies by Cagir et al. and Massomi et al. finding a female predominance among colonic ASC patients [4,5]. This difference is likely due to the relatively small size of the sample, which may skew these results. Notably, the average age of patients at the time of diagnosis in this study was 48.7 years, with most patients diagnosed prior to age 50. This is again consistent with many studies highlighting a lower average age at the time of diagnosis with colonic ASC compared to colonic adenocarcinoma [5,6]. These studies highlight the average age of diagnosis of colonic ASC as being around the sixth decade of life, although it has been identified in patients as young as 16 years of age [7]. Notably, the average patient age at diagnosis in this study is significantly younger than that reported in other studies, although this again may be due to the effects of the small sample size.

Although Cagir et al. initially reported a left-sided pre-dominance for primary sites of colonic ASC, subsequent series have found ASC to more commonly arise in the right colon. This is concurrent with this study’s findings, with just under half of patients having their primary site as the ascending colon or the cecum. Additionally, all but one patient in the cohort was diagnosed with Stage III or IV disease. Previous studies have found that 36.6–41.3% of colonic ASC patients present with Stage IV disease, which is similar to the 46.2% reported in this study [5,8]. The combination of an earlier age of presentation and a more advanced stage at the time of diagnosis associated with ASC is likely central to the poorer outcomes associated with this malignancy.

That said, the underlying pathological components may also contribute to the more aggressive nature of colonic ASC. ASC can have variable amounts of squamous and glandular components upon histology. Based on the arrangements of these components, tumors can be further classified as the collision type, in which the glandular and squamous components are distinct, or the composite type, in which these components are mixed together [9,10]. Additionally, these components can have variable morphology, including signet cells in the glandular components [11]. The specific mechanism giving rise to these multiple components is unclear. Immunohistochemical analyses have found that the squamous components of colonic ASC typically express cytokeratin (CK) 5 and 6, as well as P63, which are more classically associated with squamous cell carcinoma [12,13]. Of particular note, while human papillomavirus (HPV), especially high-risk strains 16 and 18, are associated with squamous cell oropharyngeal or anal carcinoma, studies largely fail to show evidence of HPV DNA in ASC specimens [11,13,14].

In contrast, the glandular components of colonic ASC are more likely to express CK20 and the homeobox protein CDX-2 [13,15]. While these findings might suggest two different cell populations, other immunohistochemical studies suggest that both components arise from the same progenitor cells [16]. Regardless of the underlying mechanism, the presence of squamous components may contribute to both the more aggressive behavior of these tumors and to certain paraneoplastic syndromes, such as parathyroid hormone-related protein (PTHrP)-mediated hypercalcemia that has been reported with some cases of colonic ASC [17,18,19]. Further study is needed to characterize these phenomena and the origin of the multiple components of colonic ASC. While many studies have investigated the degree of differentiation of colonic ASC on pathology, limited data exist regarding specific genetic changes. Several mutations, such as those resulting in mismatch repair (MMR) deficiency, are of clinical relevance and have been documented in cases of colonic ASC [20,21]. Most reports of MMR deficient cases of colonic ASC do not have germline mutation data available, although Duncan et al. documented a case of colonic ASC with MMR deficiency in a patient subsequently confirmed to have Lynch syndrome in germline mutation analysis [22,23]. In this study, while MMR data were not available for every patient, among those with MMR data, only one of eight had microsatellite instability, with the status unknown in the remaining five patients. While the prevalence of microsatellite instability in adenocarcinoma is difficult to quantify, one study estimated it at about 10.7% among those diagnosed with colon cancer prior to age 50 [24]. This overall prevalence is comparable to the prevalence within this cohort (12.5% among patients with known MMR genetics, 7% of all patients), which suggests similar rates of microsatellite instability in colonic ASC and colonic adenocarcinoma. It is also comparable to genetic profiling performed by Angerilli et al., which found the rate of MMR deficiency to be 4.5% among cases of colonic ASC [25].

Additionally, although whole exome genetic data were only available for a small subset of the cohort, the data that were present suggested that outside of mismatch repair deficient tumors, the mutational burden was not particularly high. The most common genes affected by mutations were genes already well established in colon cancer oncogenesis, namely KRAS, APC, and TP53. These mutations (and to a lesser degree, BRAF), have also been implicated in studies studying the genetic profiles of colonic ASC [25]. KRAS mutations have additionally been identified via genetic sequencing in several case reports of colonic ASC [26,27]. Case reports have additionally documented the presence of other notable mutations in colonic ASC, such as BRAF V600E, which is classically associated with MMR deficiency but can also (more rarely) occur in MMR-proficient tumors [28]. Our limited sample with available whole exome data, as well as the rarity of colonic ASC overall, limit drawing further conclusions regarding mutational profile, and additional, larger-scale studies remain to be conducted to fully elucidate the genetic profiles of colonic ASC and associated clinical implications.

With regards to outcomes, the 5-year overall survival rate of 38.5% (and 33.3% for Stage III and IV disease) is greater than that reported in other studies. For instance, an earlier retrospective study by Frizelle et al. identified a 24% 5-year survival rate for Stage III disease and a mean overall survival rate of 8.5 months for Stage IV disease [29]. The 5-year survival from this study may again be an artifact of the small sample size, although the reported median overall survival of 39.4 months is similar to the 35.5-month figure reported by Masoomi et al. [8]. Regardless, outcomes in this cohort were poor overall, not just in terms of survival, but also in terms of recurrence, with over half of patients showing recurrence. These findings collectively confirm prior studies highlighting poor overall outcomes with colonic ASC compared to colonic adenocarcinoma. How best to improve these poor outcomes remains unclear. Patients within this cohort received treatment largely in concordance with AJCC guidelines, although only three patients received neoadjuvant chemotherapy. A greater emphasis on neoadjuvant chemotherapy prior to surgical intervention may be of benefit for colonic ASC given its propensity to present at more advanced stages. Notably, the 2023 FOxTROT trial found that short-course neoadjuvant chemotherapy, followed by adjuvant chemotherapy post-operatively, for locally advanced colon cancer was associated with a more complete pathologic response, a lower risk of recurrence at 2 years, and improved survival versus a longer post-operative course of adjuvant chemotherapy alone [30]. Additionally, a recent study of the Surveillance, Epidemiology, and End Results (SEER) program database found that in patients with colonic squamous cell carcinoma, the administration of chemotherapy was independently associated with survival benefits and significantly improved overall survival in those who underwent surgical resection [31]. These studies suggest that a greater emphasis on chemotherapy, both adjuvant and neoadjuvant, should be considered for colonic ASC, although additional studies are needed to validate its efficacy for colonic ASC specifically.

Our study has several limitations, namely pertaining to the small sample size, which reflects the incredible rarity of the disease. As mentioned, many differences between the findings of this study and those from other studies may be artifacts due to the small sample size of thirteen patients. Additionally, the small sample size prevented direct comparison of treatments and outcomes among these patients with colonic ASC to comparable patients with colonic adenocarcinoma. This lack of context and comparison makes it more challenging to interpret our findings on patients with colonic ASC. Finally, the small size and associated lack of statistical power did not allow for analysis of whether certain interventions or tumor characteristics influenced outcomes. Again, this makes it challenging to assess what specifically about colonic ASC in this cohort resulted in such poor overall outcomes. These aforementioned areas all warrant further investigation but will also require a much larger-scale study to accomplish.

## 5. Conclusions

Colonic adenosquamous carcinoma is an extremely rare entity, making its study and characterization challenging. Here, we demonstrate the clinical properties of a cohort of patients with colonic ASC, characterized by relatively young age at the time of diagnosis, right-sided predominance, an advanced stage at the time of diagnosis, frequent recurrence, and poor overall survival. We likewise demonstrate that the genetic profile of these tumors, with regards to mismatch repair and mutation burden, is comparable to the much more common colonic adenocarcinoma. All of these add further to the limited body of literature highlighting the more aggressive nature of colonic ASC, particularly when compared to adenocarcinoma. Given the propensity to present at more advanced stages, patients with colonic ASC may benefit from neoadjuvant chemotherapy, or targeted therapy prior to surgical intervention.

Additional research will need to be conducted to directly compare comparably staged ASC to adenocarcinoma to assess outcomes and better delineate treatment recommendations.

## Figures and Tables

**Table 1:** Demographic information of patients within adenosquamous carcinoma cohort.

Age	
Mean age (yrs, ±s.d.)	48.7 ± 16.1
Age < 50 (n, %)	7 (58.3%)
Gender (n, %)	
Male	8 (61.5%)
Female	5 (38.5%)
Race (n, %)	
White	10 (76.9%)
Black	1 (7.7%)
Other/Unknown	2 (15.3%)

**Table 2:** Clinicopathologic characteristics of adenosquamous carcinoma tumors in patient cohort. Staging is based on American Joint Committee on Cancer (AJCC) Guidelines for colon cancer. (MMR = mismatch repair).

Primary Tumor Location (n, %)	
Cecum/Ascending Colon	6 (46.2%)
Transverse Colon	2 (15.4%)
Splenic Flexure	2 (15.4%)
Descending Colon	2 (15.4%)
Sigmoid Colon	1 (7.7%)
Stage at Diagnosis (n, %)	
II	1 (7.7%)
III	6 (46.2%)
IV	6 (46.2%)
Pathology (n, %)	
Moderately Differentiated	6 (46.2%)
Poorly Differentiated	7 (58.3%)
MMR status (n, %)	
Proficient	7 (58.3%)
Deficient	1 (7.7%)
Unknown	5 (38.5%)

**Table 3:** Detailed breakdown of surgical and chemotherapy interventions within adenosquamous carcinoma cohort, as well as data on recurrence, 5-year overall survival, and median survival.

*Management*	
**Surgery (n, %)**	**11** (84.6%)
Partial Colectomy	**7** (58.3%)
Total Colectomy	**3** (23.1%)
Low Anterior Resection	**1** (7.7%)
**Chemotherapy (n, %)**	**11** (84.6%)
Adjuvant only	**8** (61.5%)
Neoadjuvant only	**1** (7.7%)
Neoadjuvant and Adjuvant	**2** (15.4%)
***Clinical Outcomes***	
**Recurrence (n, %)**	**7** (58.3%)
Locoregional	**4** (30.8%)
Distant	**3** (23.1%)
**5-year overall survival**	38.5%
Stage III	33.3%
Stage IV	33.3%
**Median overall survival (months)**	39.4
Stage III	30.5
Stage IV	23.7

## Data Availability

Deidentified data are present on the institutional server.
